# Boosting protection for patients with non-acute cardiovascular disease: a focus on antithrombotic regimen (a consensus expert opinion from the Egyptian Society of Cardiology working group of thrombosis and prevention)

**DOI:** 10.1186/s43044-023-00364-3

**Published:** 2023-05-08

**Authors:** Ahmed Bendary, Bassem Zarif, Hala Mahfouz Badran, Khaled Shokry, Hamza Kabil

**Affiliations:** 1 Cardiology Department, Faculty of Medicine, Benha University, Benha, Egypt; 2 National Heart Institute, Giza, Egypt; 3 Cardiology Department, Faculty of Medicine, Menofia University, Shibin El Kom, Egypt; 4 Cardiology Department, Military Medical Academy, Cairo, Egypt; 5 Cardiology Department, Faculty of Medicine, Damietta University, Damietta, Egypt

**Keywords:** Rivaroxaban, Atherosclerosis, Risk, Egypt

## Abstract

**Background:**

Till the moment of this document writing, no Egyptian consensus is there to guide selection of additional antithrombotic in stable patients with established CVD. Despite use of lifestyle measures and statins, those patients with established CVD still face a considerable burden of residual risk.

**Main body:**

With the evolvement of evidence-based medicine, there have been a lot of recommendations to use additional antithrombotic medications to maximize protection for those patients. Accordingly, the Egyptian Society of Cardiology working group of thrombosis and prevention took the responsibility of providing an expert consensus on the current recommendations for using antithrombotic medications to maximize protection in stable patients with established CVD. For stable patients with established CVD, in addition to proper lifestyle measures and appropriate dose statins, we recommend long-term aspirin therapy. In patients who are unable to take aspirin and in those with a history of gastrointestinal bleeding, clopidogrel is a reasonable alternative.

**Conclusions:**

For some stable atherosclerotic CVD patients who are at high risk of cardiovascular events and at low risk for bleeding, a regimen of rivaroxaban and aspirin might be taken into consideration.

## Background

Patients with established coronary heart disease (CHD) or peripheral artery disease (PAD) have markedly higher risks of subsequent cardiovascular events, including myocardial infarction (MI), stroke, and death from cardiovascular disease (CVD). Therapeutic lifestyle changes of proven benefit include multiple major modifiable risk factors for CHD [elevated low-density lipoprotein (LDL) cholesterol, hypertension, smoking, obesity, physical inactivity, and diabetes] to reduce risks of future CVD events, and benefits are additive. Moreover, evidence-based doses of a high-intensity statin regardless of the baseline LDL cholesterol are also recommended. However, a high burden of residual risk remains. With the evolvement of evidence-based medicine, there have been a lot of recommendations to use additional antithrombotic medications to maximize protection for those patients. Accordingly, the Egyptian Society of Cardiology (EgSC) working group of thrombosis and prevention took the responsibility of providing an expert consensus on the current recommendations for using antithrombotic medications to maximize protection in stable patients with established CVD.

## Main text

### Populations targeted by this report

Patients with *established* cardiovascular disease (CVD) have a high risk of subsequent CVD events, including myocardial infarction (MI), stroke, and death. For all these high-risk patients, therapeutic lifestyle changes such as increased physical activity, dietary modification/weight loss, and smoking cessation are of proven benefit and improve outcomes beginning within a matter of weeks. In addition, adjunctive drug therapies of proven benefit include statins and aspirin. For the purpose of this report, we are addressing a broad group of patients who already have signs or symptoms of established CVD such as angina, transient ischemic attack, or claudication. This report is a broad overview of the approach endorsed by the EgSC working group of thrombosis and prevention to the use of additional antithrombotic medications for prevention of future CVD events in those with established CVD.

### Antiplatelet therapy

For patients with established atherosclerotic CVD, long-term aspirin therapy is recommended. Long-term antiplatelet therapy with aspirin reduces the risk of subsequent myocardial infarction (MI), stroke, and cardiovascular death among patients with chronic coronary syndromes and/or PAD. In patients who are unable to take aspirin and in those with a history of gastrointestinal bleeding, clopidogrel is a reasonable alternative.

For patients who have undergone percutaneous coronary intervention (PCI) with stenting or those who have had an acute coronary syndrome (ACS), a P_2_Y_12_ receptor blocker is added to aspirin for at least 6–12 months.

The use of dual antiplatelet therapy has been evaluated in populations other than those with ACS or those who have had PCI. In the THEMIS trial [1], 19,220 patients with chronic coronary syndrome and type 2 diabetes mellitus were randomly assigned to ticagrelor 60 mg or placebo twice per day. All patients received low-dose aspirin once per day. Patients assigned at random to ticagrelor and aspirin had a 10% lower risk of ischemic CVD events (cardiovascular death, MI, or stroke) at 40 months when compared with aspirin alone (7.7 vs. 8.5%; hazard ratio [HR] 0.90, 95% CI 0.81–0.99) but a highly significant and clinically important large increases of major bleeding (2.2 vs. 1.0%; HR 2.32, 95% CI 1.82–2.94) and intracranial hemorrhage (0.7 vs. 0.5%; HR 1.71, 95% CI 1.18–2.48). Based on these data, the US Food and Drug Administration (FDA) approved dual antiplatelet therapy for this population with or without diabetes. As in the case of all such patients, the health care provider must weigh the benefits of occlusion against the risks of bleeding for each of their patients.

In a prespecified subgroup analysis of THEMIS (THEMIS-PCI) [2] in the 11,154 patients with PCI, patients assigned to ticagrelor, and aspirin had a 15% significant decreased incidence of ischemic CVD events compared with those assigned to aspirin and placebo (7.3 vs. 8.6%; HR 0.85, 95% CI 0.74–0.97). In this subgroup, those assigned to ticagrelor and aspirin also had an over 80% significantly increased risks of major bleeding (2.0 vs. 1.1%). Intracranial hemorrhage occurred in 0.6% of both groups. In the subgroup of patients with chronic coronary syndrome and type 2 diabetes mellitus with no history of PCI, there was no apparent benefit. This subgroup analysis contributes to the formulation of the hypothesis that patients with chronic coronary syndrome and diabetes at very high ischemic risk and low bleeding risk may have a net benefit with long-term dual antiplatelet therapy with aspirin and ticagrelor. Health care providers should consider these possibilities in discussions with their patients.

### Anticoagulant therapy

For most patients with stable CAD on antiplatelet therapy, rivaroxaban 2.5 mg orally twice per day and aspirin may be considered for some stable atherosclerotic CVD patients at high risk of cardiovascular events and low risk of bleeding, based on the COMPASS trial, which is detailed below. Such patients include those with peripheral artery disease or a history of ischemic stroke, multi-vessel CAD, incomplete coronary revascularization, diabetes, patients with a body weight > 60 kg (132 pounds), prior coronary artery bypass surgery, chronic kidney disease, or multiple prior ischemic events. We *do not recommend* substituting or adding full-dose oral anticoagulant therapy to aspirin therapy in an attempt to lower the risk of subsequent CVD events.

In the COMPASS trial [3], 27,395 patients with stable CAD or peripheral artery disease were randomly assigned to rivaroxaban plus aspirin, rivaroxaban alone, or aspirin alone with a mean follow-up of 23 months. The dose of rivaroxaban in the combination arm was 2.5 mg orally twice per day; in the rivaroxaban-only arm, the dose was 5 mg orally twice per day. Compared with those assigned at random aspirin alone, patients assigned to rivaroxaban plus aspirin had a 22% significant decreases in cardiovascular mortality (1.7 vs. 2.2%; HR 0.78, 95% CI 0.64–0.96) and 49% decrease in ischemic stroke (0.7 vs. 1.4%; HR 0.51, 95% CI 0.38–0.68). There was also a possible but nonsignificant 14% reduction in MI (1.9 vs. 2.2%; HR 0.86; 95% CI 0.70–1.05). As expected, those assigned to combination therapy had a 70% significant increase in major bleeding events (3.1 vs. 1.9%; HR 1.70, 95% CI 1.40–2.05), with the gastrointestinal tract being the most common site of major bleeding. The risk of intracranial hemorrhage was comparable between the two groups. Mortality and cardiovascular outcomes were similar in the rivaroxaban-alone and aspirin-alone groups, but there were more major bleeding events in those assigned to rivaroxaban and aspirin.

In a prespecified subgroup analysis from the COMPASS trial, among nearly 7500 participants with PAD, rivaroxaban plus aspirin reduced the composite of cardiovascular death, myocardial infarction, or stroke compared with aspirin alone (HR 0.72, 95% CI 0.57–0.90) along with major adverse limb events (1 vs. 2%; HR 0.54, 95% CI 0.35–0.82). This benefit was similar for PAD patients with DM (HR 0.69, 95% CI 0.53–0.91) compared with those without DM (HR 0.69, 95% CI 0.50–0.94) [4].

In another large international trial, 6564 patients with symptomatic PAD who underwent successful lower extremity revascularization within the preceding 10 days were randomly assigned to rivaroxaban 2.5 mg twice daily plus aspirin or placebo plus aspirin (VOYAGER-PAD) [5]. Approximately 40% of patients in VOYAGER-PAD trial had DM. Clopidogrel could be administered for up to 6 months after revascularization at the discretion of the investigator and was used in 51% of participants at baseline. At a median of 28 months of follow-up (interquartile range 22–34), use of rivaroxaban plus aspirin significantly reduced the primary efficacy outcome (composite outcome of acute limb ischemia, major amputation for vascular causes, stroke, myocardial infarction or cardiovascular death) compared with placebo plus aspirin (17.3 vs. 19.9%; HR 0.85, 95% CI 0.79–0.96). There was no observed heterogeneity of treatment effect with rivaroxaban for participants with compared to without diabetes mellitus. While the incidence of thrombolysis in myocardial infarction major bleeding did not differ between groups, the incidence of International Society on Thrombosis and Haemostasis bleeding was significantly higher with rivaroxaban and aspirin than with aspirin alone (6 vs. 4%; HR 1.42, 95% CI 1.10–1.84).

The role of aspirin in patients without established CVD but at high risk, the use of aspirin plus anticoagulant therapy in patients with a specific indication for anticoagulant, such as atrial fibrillation or venous thromboembolic disease, the optimal antithrombotic strategy in patients with other reasons for anticoagulation, such as atrial fibrillation, and the role of anticoagulant therapy in secondary prevention in patients with an ACS are beyond the scope of this report.

### Who are at high bleeding risk?

Several bleeding risk scores have been developed and validated, mostly in patients receiving warfarin for atrial fibrillation. However, the authors of this report acknowledge that these scores generally do not perform significantly better than the proper clinician judgment based on close consideration of patient characteristics. As an example, in a prospective cohort of 515 patients, subjective estimates of bleeding risk made by the treating clinicians (mean clinical experience: 3 years) had similar accuracy in predicting bleeding as use of a risk score [6].

We *recommend* using major and minor criteria of high bleeding risk adopted from the COMPASS trial and proposed in Table 1 for deciding whom to treat with an additional antithrombotic medication in stable patients with established CVD.

**Table 1:** Major and minor criteria for high bleeding risk

Major	Minor
	Age ≥ 75 years
Anticipated use of long-term oral anticoagulation	
Severe or end-stage CKD (eGFR < 30 mL/min)	Moderate CKD (eGFR 30–59 mL/min)
Hemoglobin < 11 g/dL	Hemoglobin 11–12.9 g/dL for men and 11–11.9 g/dL for women
Spontaneous bleeding requiring hospitalization or transfusion in the past 6 months or at any time, if recurrent	Spontaneous bleeding requiring hospitalization or transfusion within the past 12 months not meeting the major criterion
Moderate or severe baseline thrombocytopenia (platelet count < 100 × 10^9^/L)	
Chronic bleeding diathesis	
Liver cirrhosis with portal hypertension	
	Long-term use of oral NSAIDs or steroids
Active malignancy* (excluding nonmelanoma skin cancer) within the past 12 months	
Previous spontaneous ICH (at any time)	Any ischemic stroke at any time not meeting the major criterion
Previous traumatic ICH within the past 12 months	
Presence of a bAVM	
Moderate or severe ischemic stroke^◊^ within the past 6 months	
Nondeferrable major surgery on DAPT	
Recent major surgery or major trauma within 30 days	

*CKD* chronic kidney disease, *eGFR* estimated glomerular filtration rate, *NSAID* nonsteroidal anti-inflammatory drug, *ICH* intracranial hemorrhage, *bAVM* brain arteriovenous malformation, *DAPT* dual antiplatelet therapy, *PCI* percutaneous coronary intervention

*Active malignancy is defined as diagnosis within 12 months and/or ongoing requirement for treatment (including surgery, chemotherapy, or radiotherapy)

^◊^National Institutes of Health Stroke Scale score ≥ 5

## Conclusions

For stable patients with established CVD (including those with PAD), in addition to proper lifestyle measures and appropriate dose statins, we recommend long-term aspirin therapy. Long-term antiplatelet therapy with aspirin reduces the risk of subsequent MI, stroke, and cardiovascular death among patients with chronic coronary syndromes and/or PAD. In patients who are unable to take aspirin and in those with a history of gastrointestinal bleeding, clopidogrel is a reasonable alternative.

For most patients with chronic coronary syndrome on antiplatelet therapy, we do not substitute or add a full-dose oral anticoagulant therapy to aspirin therapy. For some stable atherosclerotic CVD patients who are at high risk of cardiovascular events and at low risk of bleeding, a regimen of rivaroxaban 2.5 mg orally twice per day and aspirin should be considered.

## Data Availability

All data are available upon request from the corresponding author(s).

## Abbreviations

ACS: Acute coronary syndrome
CVD: Cardiovascular disease
EgSC: Egyptian Society of Cardiology
LDL: Low-density lipoprotein
MI: Myocardial infarction
PCI: Percutaneous coronary intervention
